# Perceptions of barriers to providing good cat care in Malaysian clinical practices

**DOI:** 10.1017/awf.2023.91

**Published:** 2023-10-16

**Authors:** Syamira Syazuana Zaini, Claire Phillips, Jill R D MacKay, Fritha Langford

**Affiliations:** 1 Royal (Dick) School of Veterinary Studies, The University of Edinburgh, Easter Bush Campus, Midlothian EH25 9RG, UK; 2 Faculty of Veterinary Medicine, Universiti Putra Malaysia (UPM), 43400 UPM, Serdang, Selangor, Malaysia; 3 Animal Science, School of Natural and Environmental Science, Newcastle University, Newcastle-upon-Tyne NE1 7RU, UK

**Keywords:** animal welfare, barriers, cat, environmental need, veterinarian, veterinary practice

## Abstract

Many veterinary practices around the world do not meet basic post-operative cat care, thereby compromising cat welfare. Understanding why the appropriate care is not always given is important. The current study used a mixed methods approach of two phases, to investigate the barriers Malaysian veterinarians face in seeking to provide good cat care in practice. Phase 1 involved a survey consisting of 14 questions which were divided into three sections (demographic details, basic management and barriers experienced by practices) and emailed to 143 Malaysian veterinarians. While for phase 2, 20 interviews were undertaken (recruited from the survey sample) to further elaborate on the results. A Thematic Analysis was conducted to extract the main barriers experienced by participants. A total of 49 veterinarians completed the survey. Over half of the respondents were senior veterinarians (i.e. those with two or more years in practice) (53.1%; n = 26) who were aware of the basic environmental provisions that cats need post-surgery such as bedding and toileting facilities (57.1%; n = 28). Cost (47%; n = 23) was the biggest restriction to good care provision. Interview findings showed that participants were aware of comfortable post-surgery environments helping recovery, but barriers were highlighted: workload factors and a lack of understanding of cat pain behaviours and associated stress. This suggested that participants had the knowledge required to provide good cat care but experienced difficulties putting this into practice. Therefore, to improve cat welfare in veterinary practice, instead of focusing purely on education, interventions to increase good cat care could include targeted elements that support behaviour change to overcome the barriers.

## Introduction

Despite research demonstrating a link between appropriate clinical settings, good animal welfare and positive clinical outcomes in cats (e.g. good environmental management can reduce the likelihood of abnormal behaviour development [Amat *et al.* 2008; Palestrini 2009; Rodan 2010; Stella *et al.* 2015], we know from personal observations and communications that many veterinary practices around the world still do not meet these basic cat care requirements.

One study explored this dichotomy by investigating the care provided to cats owned by faculty, staff, and students at two Midwest veterinary schools (Stella & Croney 2016). Most owners in the study did not provide a safe place, multiple and separated key environmental resources (such as food, water, toileting areas, scratching areas, play areas and resting or sleeping areas) as recommended as best practice for promoting the welfare needs of cats (Ellis *et al.* 2013). For example, 31% of owners placed their cat’s resting area where there was a high degree of disturbance and 35% of them did not provide a litter tray in an appropriate or private area (Stella & Croney 2016). Both of which may compromise a cat’s comfort and welfare.

Although the above study explored the cat-home setting (Stella & Croney 2016), findings have shown that even participants with a background in veterinary medicine still did not meet the environmental needs suggested by cat experts (Ellis *et al.* 2013). This could potentially reflect the management in clinical settings and understanding the relationship between veterinarians and provision of environmental needs for cat patients. Even though the key environmental resources might differ from the cat-home setting within the veterinary setting, for example, no scratching post or playing areas due to lack of space, the remaining resources such as food, water, toileting facilities and resting or sleeping areas should not be overlooked. All these basic needs are required for cats to enable them not only to express their normal behaviour but also to reduce their stress levels and provide a place for comfortable rest (Crouse *et al.* 1995), which offers freedom from fear and distress (Stella *et al.* 2011; Amat *et al.* 2016), especially for the recovery of patients having undergone a surgical procedure. As some behavioural indicators of stress and pain overlap, minimal stress experienced by cat patients may help veterinary professionals to better understand and recognise pain behaviours in cats. However, many veterinary practices around the world do not meet basic post-operative cat care, thereby compromising cat welfare.

There may be a number of factors involved in a poor post-operative environment; for example, the socioeconomic status and the level of animal welfare awareness in the country in question. Previous studies in the United States and Canada have reported clients’ economic limitations as the main factor contributing to veterinarians’ inability to provide the desired care for patients (for example, Kipperman *et al.* 2017). Another study (Kinnison *et al.* 2015), conducted in the UK, identified a few challenges to interprofessional team working which may cause clinical errors in veterinary practices such as a lack of time, a reliance on part-time staff leading to frequent handovers, branch differences and also individual veterinary surgeon work preferences (Kinnison *et al.* 2015). Taken together, both studies illustrate that a lack of self-confidence around client conversations combined with ineffective action plans in practices and difficulties in staff management could all create underlying social-cognitive factors that could impact cat care.

However, these possible limitations or barriers from the UK, US and Canadian veterinary practices may or may not be present in other countries. As, according to Thornton (2013), an increase in country income sees an increase in concern for animal welfare within the country’s veterinary practices. There have been no studies as yet exploring the barriers Malaysian veterinarians face as they seek to provide good post-operative care for cats in practices.

The study aimed to understand the specific barriers experienced by Malaysian small animal veterinarians in seeking to provide good clinical care for post-operative cats. A mixed-method study; an explanatory sequential design will be used first by quantitative survey to aim to (1) investigate the barriers affecting the post-operative management of cats. Subsequent to the survey, a qualitative semi-structured interview aimed to investigate (2) the context in which these barriers are encountered and (3) how veterinarians understand post-operative management and cat pain assessment relationships.

## Materials and methods

### Ethical approval, consent and data management

The study was approved by the Human Ethics Research Committee (HERC) on 25^th^ January 2017, Royal (Dick) School of Veterinary Studies, University of Edinburgh: HERC approved project number HERC_154_17. The project also had ethical approval from the Malaysian Medical Research and Ethics Committee (MREC): approved project number nmrr-17-672-35076. All the responses remained anonymous and were not shared with any third party. They were stored on a secure server which was held in relation to the UK Data Protection Act in the United Kingdom. An informed consent document was developed, approved and used for the study so that all participants were made aware of the study’s purpose, understood how their data were to be used and handled and understood they could withdraw their data right up until the date of completion of the PhD thesis.

### Study design

**Figure 1.**
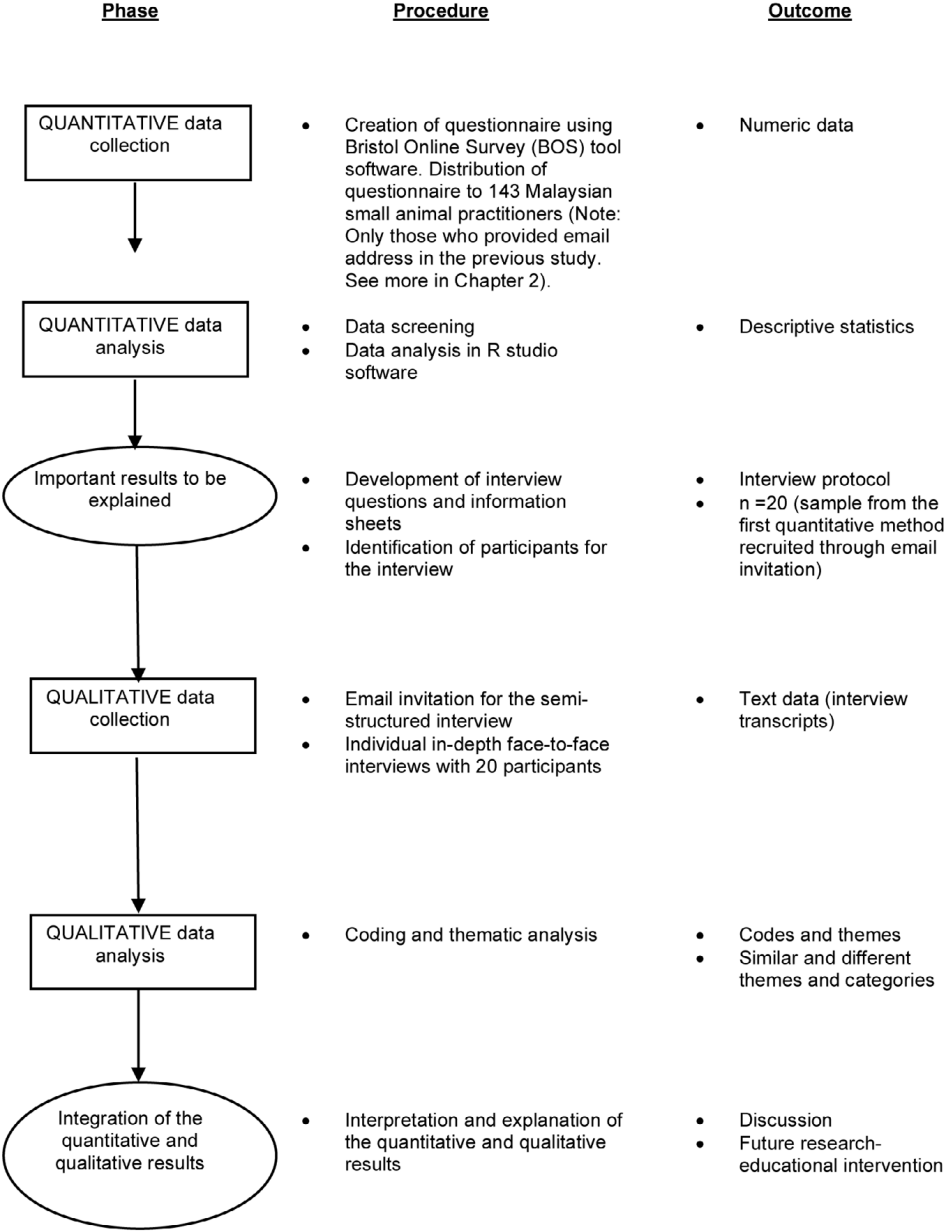
Diagram of the explanatory sequential mixed-methods design as adapted for the current experiment (Creswell *et al.* 2003).

This mixed-methods study used an explanatory sequential design that firstly involved using quantitative methods, followed by qualitative methods (see Figure 1). In the first phase, questionnaire data were collected from small animal veterinarians to examine the barriers affecting the post-operative management of cats in practices. In the second phase, the qualitative semi-structured interview was used to help explain the quantitative results in more depth, so as to understand the context in which these barriers were encountered; such barriers having been indicated in the first phase of this study. The qualitative method also helped identify the extent to which veterinarians understood the relationship between reduced environmental management and post-operative pain in cats.

### First stage: Quantitative data collection

#### Questionnaire development

The questionnaire was developed to investigate the barriers Malaysian veterinarians face in implementing good post-operative care of cats in practices. Since there is a lack of evidence and reporting about these barriers – especially in veterinary practices – some questionnaire items developed were based on Kinnison *et al.* (2015) and Kipperman *et al.* (2017) (Table 1). Statements were altered in order to ensure that the presentation did not create confusion for the Malaysian veterinarians. The remaining items were specifically developed for the current study. For example, Standard Operating Procedure (SOP) questions were included in this questionnaire with the aim of investigating the availability of any guidelines that assist veterinary professionals in providing good cat care in practice.

**Table 1.** Summary of questionnaire

Section	Questions	Type	Reference
Veterinary demographic	Are you a small animal practitioner (Mostly handling cats and/or dogs)?	Multiple choice (single answer) question	−
	What is your role in your practice?		−
	Are you aware of Standard Operating Procedures (SOP) in your clinical practice?		−
	What is your role in these reviews of clinical guidelines or (SOPs) for clinical practice?		−
Attitude to cat pain and welfare	Attitudes to cat pain and welfare: I can recognise when a cat is in discomfort I feel uncomfortable if I see a cat in pain I feel sorry for a cat that is feeling discomfort I know that freedom from discomfort, pain and injuries is important for cats I am aware that freedom from discomfort, pain and injuries is part of cat welfare	Participants rated 4 questions on a 1-4 Likert-like scale (1 = Does not describe me at all, 2 = Describes me little bit, 3 = Describes me well, 4 = Describes me very well)	−
Basic management of cat patients in practices	What do you currently provide in the recovering cat’s cage (After full anaesthetic recovery and the cat is able to walk unaided)? Basic cat resources, e.g. bedding, litter tray, feed bowl, water bowl, hiding box, cat tree, toy	Multiple choice (multiple answer) question	−
	Is there anything you would like to provide but are unable to, or may not be allowed to in your practice?	Multiple choice (single answer) question	−
	What other items would you like to add to the cage but are unable or may not be allowed to do for your recovering cat patient (in a cage)?	Multiple choice (multiple answer) question	−
	What are the main reasons for not being able to add extra items to cages of recovering cat patients? e.g. The reasons for not being able to add extra items to cages of recovering feline patients; cost client wishes busy	Multiple choice (multiple answer) question	- Kipperman *et al.* 2017 - Kipperman *et al.* 2017 - Kinnison *et al.* 2015
	What is the main reason for not adding anything extra to the cage? Cost Takes too much time	Multiple choice (multiple answer) question	- Kipperman *et al.* 2017 - Kinnison *et al.* 2015
	What is the most effective method of ensuring the review and updating of Clinical Protocols or Standard Operating Procedures (SOPs) in clinical practice?	Open-ended question	−
	Do you have any question, comment or opinion on basic management for the cat patient?	Open-ended question	−

The questionnaire included a total of 14 questions, contained in three sections (see Table 1). The questionnaire was developed using the online software tool, JISC Online surveys. The questionnaire was in English since the majority of respondents who had agreed to be contacted via email were from a group of English-speaking survey respondents that had been recruited from previous study (Zaini *et al.* 2022).

#### Pilot questionnaire

The questionnaire was pilot-tested on five small animal practitioners in Malaysia. The pilot test indicated that a clear definition of SOP should be provided in the early stages of the questionnaire. In addition, SSZ also added an extra question to the demographic section regarding whether or not the participants had SOP in their practices. Pilot tests reported no issues related to time (e.g. it took 10 min to complete the questionnaire) for respondents. The final questionnaire was made available for eight weeks from 6^th^ of December 2017 to 6^th^ of February 2018.

### Second stage: Quantitative data analysis

All the results were collected and downloaded from the JISC online survey into Microsoft Excel®. The findings from the study were kept in the strictest confidence and with the highest levels of security as required by the UK Data Protection Act (1998), as amended by the General Data Protection Regulation (2018). All data held locally in Edinburgh were securely destroyed at the completion of the PhD project. Prior to analysis, the data were screened for missing values. There were no incomplete responses used, as the survey software JISC does not allow for the collection of incomplete data. All variations shown in the N for each question are related to the numbers of participants that responded to dependent follow-on questions. Complete data were further analysed descriptively using frequency counts in R studio, using R version number 3.4.3 (R Core Team 2017). The following packages were used; Likert (Bryer *et al.* 2016) and tidyverse (Hadley 2017).

### Third stage: Qualitative data collection (semi-structured interview)

#### Participant recruitment

A total of 20 out of 49 participants were recruited between February 2018 and March 2018 from a list of respondents to the barrier questionnaire. The targeted participants were those that reported that they mainly practiced as small animal practitioners in Malaysia. Those that agreed to participate were contacted via email and further details regarding the interview and an informed consent form were emailed to participants. However, some participants were contacted directly by SSZ as they were her veterinary colleagues.

#### Development of interview protocol

There were a total of seven open-ended questions to be discussed with participants (Table 2). The interview was undertaken by SSZ at or close to the practice in question and each was given an information sheet and a consent form beforehand and it was made clear they could withdraw at any time. The aims of the interview were made clear as was the fact that the entire conversation would be audio-recorded. Participants were invited to discuss their clinical practice and their role therein. The questions were focused on basic cat post-operative management and the clinicians’ experience in terms of the provision of good care. Additionally, a number of prompt questions were designed to ascertain the barriers faced in terms of providing good management of cat patient welfare, as well as the connection between bedding and post-operative pain assessment in cats and the specific motivations that drive the veterinarians to improve cat welfare in practices. Questions were given in English (but respondents could answer either in English or Bahasa Malaysia [Malay]) and SSZ was able to communicate in both languages which minimised the risk of misunderstandings.

**Table 2.** Summary of semi-structured interview questions

No.	Questions
1.	Could you tell me a bit about your practice?
2.	Tell me what do you do in your clinical practice for cats during post-operative recovery?
3.	From your experience, tell me about what do you do when a cat comes out after surgery?
4.	Do you ever find any difficulties to provide bedding for cat during recovery?
5.	Do you think there is a relationship between providing bedding and post-operative pain in cats?
6.	Do you think cat comfort in the clinical practice needs improving?
7.	In your opinion, what motivations are there driving veterinarians to improve cat welfare?

### Fourth stage: Qualitative data analysis

Interviews were recorded on a Digital Voice Recorder by EVIDA L60 and recordings sent for external transcription (SSZ compared the transcripts to original recordings to ensure accuracy). Back-translation was necessary for certain recordings since a number of participants responded in the Bahasa Malaysia (Malay) language. Data were formatted by hand to facilitate further analysis and analysis (e.g. extraction of the main barriers) conducted through iterative Thematic Analysis until data saturation was reached. Primarily, SSZ read and re-read the transcripts in order to increase familiarity with the data before they were coded initially using topics derived from the interview guide. The data were rearranged according to codes and as themes were drawn out from the data, they were discussed, refined and sense-checked with a co-author (JRDM) to construct a codebook (Table 3) which was then applied to all transcripts. In order to ensure validity and consistency of interpretation, an audit was then conducted by a further co-author (FL). Lastly, final themes were evaluated by the multidisciplinary team to ensure that each was distinct, meaningful and relevant to the research questions that are stated in the aims of this paper (Patton 2002). All the transcriptions and audio recordings were kept securely in files at the University of Edinburgh, UK.

**Table 3.** The codebook of the list of barriers (themes and sub-themes) experienced by veterinarians in clinical practice

Themes	Sub-themes
Theme 1: Financial constraints including clients’ economic limitations	Restricted physical resources. Restrictions on employing more staff. Restrictions which impede good management. Clients’ economic limitations.
Theme 2: Inadequate practice management skills	Multiple roles. Standardising Standard Operating Procedures (SOP). Improving management. Productive deployment of staff and efficient use of time.
Theme 3: An emphasis on cleanliness and sterility in the cage	Veterinarians’ perceptions. Did not offer 24-h service.
Theme 4: Views on the importance of bedding for reducing of post-operative stress in cats and the relationship with pain recognition	Provide comfort and warmth. Provide a safe and secure place. Subjective and individual variations. Requirement for more management presentation, no effect on post-surgery cats.

## Results

### Response rate

A total of 49 (34.3%) out of 143 veterinarians completed the questionnaire, and 20 semi-structured interviewed were conducted.

### Demographic findings

Of the respondents, 79.6% (n = 39 [questionnaire]) and 45% (n = 9 [interview]) claimed they were small animal veterinarians who were mostly handling cats and/or dogs. Most of the questionnaire (53.1%; n = 26/49) and interview (60%; n = 12/20) respondents were senior veterinarians (i.e. a veterinarian who had been in practice for two years or more) with the remainder veterinarians with less than two years in practice (i.e. ‘junior veterinarian’).

### Attitudes to cat welfare

**Figure 2.**
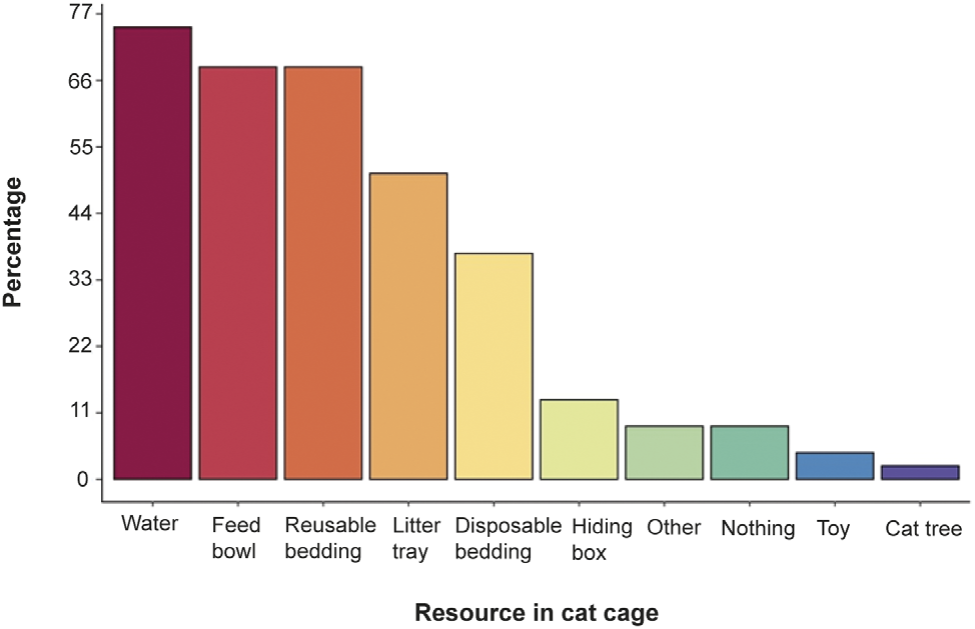
Percentage of basic resources available for post-OVH cats as reported by participants.

There were five different items (see Table 1) developed in the second part of the questionnaire to measure respondents’ attitudes towards cat welfare (Figure 2). Those items used a four-point Likert-like scale. All 49 respondents were aware of, and acknowledged, the importance of freedom from pain and discomfort in cats. Similarly, all 20 veterinarians interviewed agreed that bedding provides comfortable post-operative recovery to their cat patient.

*“Bedding definitely improves cat comfort. Especially in this cat you see here, we put in towels, we put in heater as well.”* [D083]

### Cat pain management in clinical practices

When asked about the routine of cat cage management for post-ovariohysterectomy (OVH) especially once cats have awoken and can walk unaided, a number of respondents claimed to provide such patients with their basic environmental needs, including reusable bedding (see Figure 2). Slightly over ten percent (10.2%; n = 4) claimed that nothing was provided post-OVH. Respondents who selected ‘other’ were asked to list what other resources were available for post-OVH cats and these included a heating pad, a heating lamp as well as a warm bag, and blanket.

### Reasons preventing the provision of extra resources

Of the questionnaire respondents, 42.9% (n = 21/49) wanted to provide extra resources to post-OVH cats such as a hiding place (76.2%; n = 16/21), reusable bedding (28.6%; n = 6/21), toys (14.3%; n = 3/21) or a cat tree (4.8%; n = 1/21). However, close to half of them stated that cost (47%, n = 23) was prohibitive. Other factors hindering the provision of these extra resources included the SOP which applied in the practice (6%; n = 3/48), client’s wishes (12%; n = 6/48), as well as the opinions of senior and peer veterinarians who do not deem such extra resources as being necessary (6%; n = 3/48).

Since this question was considered key to a future educational intervention study, questionnaire respondents were asked to provide details via an open question as regards their reasons for not being able to provide the cat cage with extra resources. Some of the reasons offered were as follows:

*“Limited space in the cage.”*

*“Not enough manpower to clean, cages not big enough.”*

*“Safety purposes during the recovery period. When the cat is stable, everything will be put in place accordingly.”*

### Reasons for not providing additional resources

**Figure 3.**
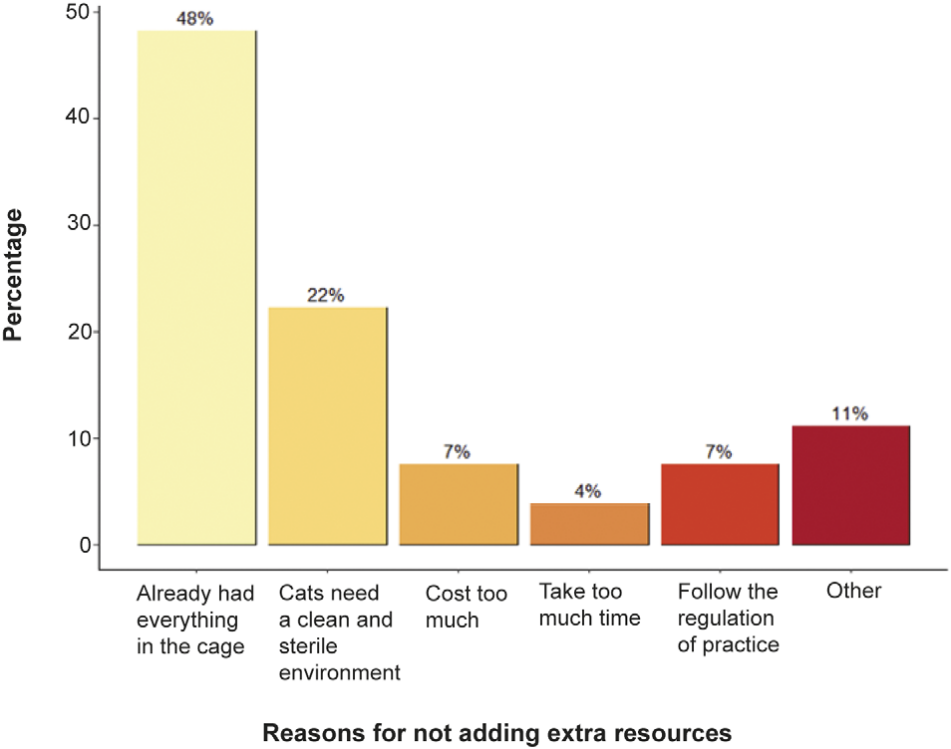
Respondents’ (n = 27 responses; because it was a multiple-choice question) reasons not providing additional resources for post-operative cats.

In the questionnaire, 57.1% (n = 28/49) claimed that they did not plan to add anything for cat patients because 48% of respondents (n = 13/27) said they had already provided all the basic resources (e.g. comfort, food, water, a hiding place, and toileting) in the cat cage (Figure 3).

### Challenges in clinical practice

In-depth explanation with regard to the barriers experienced by participants as regards the specific type of environmental need (e.g. providing comfortable bedding) were further explored through the semi-structured interviews. Provision of comfortable bedding to cat patients is considered important for comfort and welfare (Crouse *et al.* 1995), especially for those patients recovering from a surgical procedure. Although all interview participants (n = 20) were in accordance that cat bedding was important, ten also claimed that not all cats are provided with bedding in their clinic. Certain specific barriers were identified by veterinarians as to why their use of bedding was limited and not provided to all cats in practices. Therefore, the interview results yield four main themes, each representing distinct experience of barriers to providing good clinical care for cats: (i) financial constraints, including clients’ economic limitations; (ii) staffing and workload factors; (iii) an emphasis on cleanliness and sterility in the cage; and (iv) views on the importance of bedding for reducing of post-operative stress in cats and the relationship with pain recognition.

### Financial constraints including clients’ economic limitations

Lack of physical resources (e.g. limited space in cat cage due to the small size of cage, insufficient bedding supplies) was one of the barriers experienced by participants (n = 14/20) to having proper practice management for cat patients. This result was consistent with the 47% of questionnaire participants who nominated cost as a barrier to providing extra resources for cat patients. Participants claimed that having good financial support could help them to improve the welfare of cat patients in clinics.

*“For that, the first thing is the cages are costly, and secondly, we also don’t have much space if we want to order many, so we try to as much as possible to provide a comfortable space, if we can’t, then there is nothing we can do. Because to get that kind of cage, we have to consider the high costing and also it takes up a lot of space and we also use the room for storage, so there is not much space”* [D101].

*“Maybe it’s a bit, sometimes it’s a bit costly of course, I mean there is an increase in cost in providing those kinds of thing like bedding…”* [D122].

*“…we have quite many cats each day for neutering. So, we are not able to provide bedding for each one of them, as it will cost a lot…”* [D081].

In general, all the veterinarians interviewed employed a limited number of staff in their practices. As a result, they were struggling to consistently manage animal welfare.

*“It’s just that the cost of labour. Sometimes when there is, sometimes when we short of staff, and not able to wash very regularly…”* [D071].

*“…another thing is staffing. Because when you want to monitor, you have to have an extra, I mean the person needs to be there all the time”* [D086].

In order to maintain cleanliness and sterility, bedding should not be reused for other patients, however, clean bedding is not readily available when required because there are insufficient cleaning facilities in practices; for example, washing machines and drying spaces. The absence of a washing machine may mean that there is not enough available clean bedding. As a result, some veterinarians (n = 5/20) reported using their own washing machines or self-washer machines to get all the bedding cleaned; however, this can effectively reduce the supply of available bedding where the number of patients on a given day is particularly high.

*“But I think in terms of management and cost, to wash it that often and to control with the bio-security, I think that will lead to higher cost”* [D063].

Participants reported using alternative forms of bedding such as disposable materials (e.g. newspapers). However, changes in media consumption have meant that printed materials are scarcer. Also, participants appeared to want to be environmentally friendly; quotes from two of the participants.

*“Cost is definitely an influence. Definitely, as you can see, newspapers will be the cheapest to obtain, but at the same time, because a lot of people are going on electronic media, so newspaper is getting harder and harder to come by, so the absorbable pads are things that we need to purchase. In fact, the newspapers that you see here, are purchased by the shelter as well. It’s not donated; it’s purchased, because we need more regular supplies. So, it’s all cost”* [D084].

*“Now, why I don’t like using these disposable things too much, like for me I try to avoid using disposable hand towels and all that because I think that is not planet-friendly, environmentally friendly”* [D072].

Veterinarians reported that they needed to keep their prices down, so that their clients could afford to pay for them. In order to reduce the price of certain surgical procedures such as neutering, veterinarians did not provide a number of the resources required for good cat welfare, unless absolutely necessary, as any added resources cost would have raised the price of the procedure for the client.

*“It is not an urban area. People are very concerned about cost. So, we try to manage with a very low price that people can afford”* [D061].

*“Also, our neutering cost is very low, so we only provide what is necessary”* [D073].

### Staffing and workload factors

Many veterinarians revealed that they held more than one role within the practice. Not only are they the sole decision-maker for the patients but also have an administrative role, managing the practice, monitoring and controlling staff, marketing duties and dealing with clients. Performing multiple roles results in an increased workload and insufficient time for other commitments, especially those pertaining to patients, as reported here.

*“Basically, I work as a vet, mainly as a vet, and I also work on the administration side and also managing the flow of the clinic”* [D086].

*“…the welfare, just that sometimes, they’ve been dragging with too much workload and everything, and then they want everything to work fast. Sometimes, they just tend to forget…”* [D121].

SOP are designed to provide a structure so that everyone in a specific practice knows what they are supposed to be doing for any given task. In the questionnaire, 95.9% (n = 47) were aware of the availability of SOP in their practice. When asked about their role in reviewing the SOP, over half (63.3%; n = 31) reported that they contribute to protocol reviews when needed and that their ideas were listened to. None of the respondents expressed a lack of interest when it came to involvement in reviewing SOP (Table 4).

**Table 4.** Role in reviewing SOPs in clinical practice

Statements	Percentage of total respondents(n = total)
I contribute to protocol reviews when needed, and my ideas are usually listened to.	63.3% (31)
I do not contribute to protocol reviews because I am not asked to.	16.3% (8)
I contribute to protocol reviews when needed, but my ideas are rarely listened to.	10.2% (5)
I do not contribute to protocol reviews because it is not necessary for my role.	4.1% (2)
My practice does not have any clinical protocols or SOPs.	4.1% (2)
I do not know if we have this review process in my practice.	2.0% (1)
I do not contribute to protocol reviews because I am not interested in this task.	0

A factor reported by almost all veterinarians interviewed was the lack of standardisation in clinical protocols, especially concerning post-operative cat care management. This finding reflected the questionnaire responses, i.e. only 7% (n = 2/27; Figure 3) of participants followed the SOP. One possible explanation for failing to add resources in cat cages could be the lack of or a poorly structured management plan. Generally speaking, veterinarians tend to conform with protocols they perceive appropriate for the situation and conditions they encounter in their clinical practice. In addition, two of the veterinarians claimed not to provide any bedding to cats since it was not practice policy.

*“Cost? It doesn’t cost much. I think, it’s just something that we don’t practice here. But again, I said it depends on the condition. If need, we will”* [D082].

Many veterinarians also expressed difficulty with the enabling of an efficient managerial system that could be applied in practices. Many noted that staff shortages present obstacles for the efficient management of a veterinary practice.

*“My staff have been told to keep an eye on the cats here while they are recovering, and at the same time, they have other duties like preparing the instruments, preparing the animals, and doing the cleaning and so on. So, everybody is in charge of animal welfare at every stage and I think it’s not a matter of cost I would say, the cost is very minimal.”*

*“It’s just a matter of how effective you can use some of the material you have and how effective you can use the staff time that you have”* [D083].

Almost all the interviewed participants wanted a more manageable workload.

*“Of course, we always try to do things that don’t create more work for ourselves. You don’t want to create more work for myself or my colleagues”* [D072].

*“…we have to make sure the staff know what to do, because now, I know the workload increases a little bit… we have to educate and train our staff for that, because bedding I think is still going to be OK…”* [D121].

Some of the veterinarians were concerned that providing more than the bare minimum of resources for cat patients would lead to their workload becoming heavier and individual tasks taking longer to complete. Consequently, some veterinarians did not give their cat patients bedding or a litter tray. They believed it to be easier to clean a cat cage when the cat urinates directly onto the tray which is placed under the wire cage.

*“…so that is why we just leave the cage empty without anything, so the urine will just flow to the bottom of the cage….It is not to say that expensive, but we prefer to just let the cat urine flow to the bottom of the cage….It is much easier that way, as compared to if they defaecate or urinate on the towel…. It is like I said, we feel it is easier, for instance, if we do surgery on the ventral side, it will be easier to manage if there is no bedding soaked with urine”* [D073].

### An emphasis on cleanliness and sterility in the cage

These were deemed crucial by a number of the interviewed veterinarians as well as those completing questionnaires where one of the reasons offered for not providing extra resources was related to the importance of hygiene, especially post-operatively (22.2%; n = 6/28). The claim was that inclusion of bedding ran the risk of urine or faeces becoming trapped in the surgical incision, thus increasing the risk of post-operative infection in cats, as well as unsanitary.

*“And, sometimes, why we don’t really put the bedding in all situations, because sometimes during the recovery process, they will pee and poo… we don’t want the stool or urine to dirty the body”* [D122].

*“As we are using the metal cages, if we use bedding, we are afraid that it may contaminate, especially for the male cats… we don’t put it in as it may cause infection at the place of the surgery”* [D081].

As most veterinary practices did not offer a 24-h service it follows that cat patients were not monitored 24 h a day. This adds to the difficulty of maintaining cleanliness for the cat patients. In those situations where veterinarians would not be able to clean bedding, they would be unable to prevent patients becoming soiled which could lead to an increase in the chances of the surgical wound getting. This was clarified by the veterinarians D072.

*“…because one of my limitations also is our clinic, we are not 24 hours”* [D072].

### Views on the importance of bedding for reducing of post-operative stress in cats and the relationship with pain recognition

Veterinarians were asked about the relationships between reducing stress and whether or not this would impact pain recognition. They were asked specifically about the relationship between bedding and post-operative pain in cats, and a variety of different answers emerged. Some claimed the relationship to be subjective, with insufficient scientific evidence for a definitive outcome, or that the relationship depended on the specific cat’s behaviour (and was therefore subject to individual variation). Some were of the opinion that cats do not necessarily require bedding since they had observed some not finding it comforting.

*“… sometimes the cats are not used to the pads, sometimes they will scratch it, and then tear it off, and destroy it, so it’s not nice too”* [D122].

Most interviewees felt cats need to be comfortable, to have a safe and secure place during recovery. This is because different cats show different tolerances to anaesthetics meaning there is a possibility of rough recovery leading to self-injury if no cushion or bedding is in place to offer protection.

*“So it’s more for them to prevent traumatising themselves. So that is why the towel is in so they won’t knock themselves”* [D072].

*“The reason we put the green mat in is that usually the cat will be drowsy and may get hurt or get their legs caught between the bars or rub their wound on the cage and will break down. That has happened before. So we don’t want that to happen”* [D102].

However, some participants felt bedding was not necessary for cat patients because they preferred other resources, such as a litter tray.

*“But here our standard is just the normal cage which is just enough and if we put bedding inside, usually the cat will not sit on the bedding, they will instead sit in the litter tray. So, we don’t want them to sit in the litter tray with all the urine, as it is dirtier. So usually, for post-operative, we rarely provide a litter tray. We will just let the urine flow to the bottom. Even if they defaecate, we will just clean it, unless there are babies”* [D073].

*“Well, some of them like to sit in a cosy area. They feel very comfortable. But some will not sleep on the towel or bedding area. They will poo there or they will urinate there. Some don’t, some will do it away from that sleeping area”* [D061].

*“Because sometimes if there is under-pad also it may tear or overturn”* [D122].

Most of them stated that the use of bedding usually depended on the type of medical condition or surgical procedure each cat was undergoing (n = 7/20). For the major types of surgical procedures, such as orthopaedic surgery, bedding was seen as compulsory during recovery.

*“Not all. Depends, depends… Usually for surgery that related to such as amputation, and then wound, wound dressing, or something big, anaesthetic, so we will provide the bedding. But, usually after surgery such as neutering, usually we will not, because sometimes they are not so in painful condition, I mean in my opinion…”* [D122].

*“And, usually, it will depend on the surgery. If it is a routine surgery, we will not provide* [bedding]*”* [D073].

There was a difference in the way participants provided bedding, depending on the background of the cat. For example, a few participants said that the provision of environmental needs was dependent upon the individual cat’s housing management or domestic lifestyle.

*“I think, it more depends on their lifestyle. Some cats are fully indoor cats. We see the owners providing them a blanket even when they sleep. But if it is an outdoor cat who is allowed to move around outside, then usually they have the habit of rolling themselves on the roadside and all that, so those cats can be easily managed. They are not concerned whether there is any bedding or anything in the cage”* [D073].

Some participants noted that bedding could pose a risk to cat patients.

*“Because, sometimes, if there are animals that are thrashing against the cage and we put bedding, it will become more difficult for it, and may hinder and they can sometimes get caught up in the towel”* [D062].

Veterinarians stated bedding was an issue for the management process and that it did not directly reduce post-operative pain in cats:

*“So, I think it* [bedding] *provides some comfort, but I don’t think it’s related much to pain, as in the surgery”* [D091].

*“Providing bedding is more to management and not really related to post-operative”* [D062].

A number of participants felt that one reason for providing comfortable bedding to post-operative cat patients was to improve their welfare.

*“It’s* [bedding] *just more for their welfare while they are boarding or hospitalised. So, during post-operative, it’s* [bedding] *just to provide comfort during their recovery. That’s all”* [D062].

*“I think it* [bedding] *does have an effect, and I think the more comfortable you are during recovery, it’s also better for animal welfare”* [D084].

However, one participant was of the opinion that cat patients did not always require bedding as an environmental need.

*“I’ve never thought a cat needs bedding. If the patient* [cat] *needs bedding, we use it”* [D072].

## Discussion

The current study provides an insight into the barriers faced by Malaysian veterinarians when it comes to the provision of good management for post-OVH cats in cages. It revealed a variety of reasons for participants not sometimes being able to meet their cats’ basic environmental requirements. The main factor quoted was cost which is not unexpected since economic status is often reported as a reason for a lack of provision for cat welfare in developing countries (Thornton 2013) due to the conflict between maximising profits and maximising animal welfare (McInerney 2004). Unfortunately, the specific details of financial costings were too vague to be further explained through the quantitative method (i.e. the questionnaire). The explanation may be that clients have limited funds (Kipperman *et al.* 2017; Hardefeldt *et al.* 2018), or that resources such as drugs (Lascelles *et al.* 1999), or staff recruitment (Kinnison *et al.* 2015) were prohibitively high. From the short interviews, we discussed the meaning of ‘cost’ as well as other barriers which have been discussed in four separate themes (i.e. financial constraints including client’s economic limitations, workload and staffing factors, cleanliness and sterility in cat cages and the link between reducing stress and recognition of pain).

The financial barriers to purchasing physical resources were not only recognised in Malaysia, countries such as Brazil (Lorena *et al.* 2014) and Colombia (Morales-Vallecilla *et al.* 2019) also faced a similar situation. Those studies showed that veterinarians could not afford to purchase analgesic drugs for small animal patients, which was a barrier to prescribing perioperative analgesia. In the current study, cost constraints often involved purchasing physical resources (e.g. bedding, a washing machine). It is necessary for veterinary practices to find a cost-effective alternative. For example, veterinarians and staff could be asked to donate their old clothes for use as bedding.

Another aspect described in interviews was that of clients’ economic limitations. For example, respondents claimed to be worried about charging their client, lest they would be unable to afford to pay the bill. Educating clients regarding the importance of sustaining normal cat behaviour, especially in the novel environment, would also facilitate cat welfare. This could be done by asking the client to bring bedding or clothes for their pet which would help to maintain the familiar smell and scents of home for the cats (Ellis *et al.* 2013). This could also be a cost-effective way of helping overcome the clients’ economic limitations.

In addition, there were other aspects of clients’ economic limitations that influenced the management of cat patients. For practices based in small, less affluent towns, it was crucial that consultation and treatment fees were set at an affordable price and clients were offered treatments which veterinarians deemed were affordable. However, cheaper treatments may equate to poorer standards of cat care management since these may be concerned more with reducing cats’ pain and suffering during surgery than welfare overall. Similarly, in other countries, the financial status of the client has been recognised as a common barrier to providing good cat care in veterinary practices. Veterinarians in Canada and the USA described difficulties in providing a high quality of pet care due to their clients’ economic limitations (Kipperman *et al.* 2017). In Ireland, the Magalhães-Sant’Ana *et al.* (2017) study found veterinarians were forced to consider what their clients would be willing to spend before referral to other practices, even if they felt that their client might not be concerned about the cost. A productive discussion with clients regarding such costs is necessary because the financial burdens of medical care can cause a great deal of stress to clients. Financial assistance such as credit services to assist in covering the up-front cost of patient care could be offered to address these economic limitations (Kipperman *et al.* 2017).

Financial constraints not only involved purchasing resources, but also employing more staff, and some veterinarians observed their practices were particularly small. The majority of participants claimed that they had between one and three staff to manage their practices. Having low staff numbers with high workloads is potentially disruptive to managing practices, leading to less time devoted to patients. Ideally, having at least one to two qualified veterinary nurse(s) at a veterinary practice can reduce the workload for veterinarians, enabling them, for example, to focus on consultations, case diagnoses and treatments while veterinary nurses provided the care. However, there remains a paucity of literature on this as regards to Malaysian veterinary practices and so further work is required to create a fuller understanding of these relationships.

Here, most participants were concerned about efficient use of staff time in order to manage and maintain cat welfare in veterinary practices. However, the appropriate handling of cat patients was hampered by a combination of staff shortages and inadequately sized cat cages. As a result, a number of participants did not provide bedding to cat patients in order to reduce staff workloads, especially in terms of reducing the need for extra washing and cleaning of bedding. These findings may reflect the current veterinary training curriculum in Malaysia. It is important to evaluate the efficacy of veterinary business and veterinary entrepreneurship courses in the Malaysian veterinary curriculum especially as regards a potential revision of course content, method of delivery method and learning outcomes. Knowledge of practice management should be advocated in the Malaysian veterinary curriculum. Currently, no specific training exists regarding this subject for Malaysian veterinary students that might enable development of a skill set, boosting confidence in this field.

In this study, most participants wanted cleaner environments for cat patients yet bedding was not considered important. For example, some felt bedding would increase the risk of infection at the surgical site. This suggests that such is the focus on cleanliness that the importance of comfort for cat welfare can be overlooked. As such, participants failed to acknowledge the importance of maintaining normal behaviour for cat welfare. As reported elsewhere, studies suggest that cat pain has been undertreated (Hugonnard *et al.* 2004; Perret-Gentil *et al.* 2014; Beswick *et al.* 2016) and the point is reinforced here. It is possible that cat pain is not treated because cat behaviour is being misinterpreted, which itself may be due to cats being kept in conditions that do not meet their welfare needs. It must be recognised that the use of barren cages in veterinary practices can harm cat welfare. It is essential that participants acknowledge this as its recognition could help motivate a shift in behaviour towards improving the welfare of cats. A failure to properly acknowledge the effects of barren cages on cat welfare will reduce the likelihood of change occurring (Schwarzer 2016).

Although participants claimed to provide comfortable bedding to cat patients, this was not the case for all cat patients. It did not include those undergoing routine surgeries such as neutering whereas it did for major procedures such as orthopaedic surgery, or those subject to certain kinds of housing management (e.g. indoor, outdoor, and stray cats). As such, we should take the positive outcome of the questionnaire results (i.e. that most respondents did provide at least one resource) with some caution. Even though the questionnaire did ask specifically about post-OVH cat patients, it is likely that respondents generalised their answers to encompass all cat patients.

In cases of cats with serious or life-threatening ailments such as chronic kidney disease, traumatic injuries (e.g. those hit by a car or suffering from high-rise syndrome) or kittens recently born at the practice, these were more likely to not only receive additional attention but also have their needs (e.g. for a comfortable resting area and isolated cat cage) met. When such care is compared with those in routine cases or undergoing routine surgeries such as neutering, most participants found neutering to be a simple and straightforward procedure. Therefore, based on participants’ experiences, there is a clear perception that neutering was not a serious surgical procedure, and that it was less painful than orthopaedic surgery. For that reason, providing cats with a comfortable environment was often viewed by interviewees as less important for post-OVH cats than for cats undergoing major surgeries. This is an interesting finding since from a veterinarian perspective OVH may be viewed as routine (Van Goethem *et al.* 2006) however for the cat in question the procedure is far from routine, i.e. the veterinary point of view may fail to take into account the cats’ perspective. This also suggests that most of the study participants were more focused on physical medicine skills than the psychological aspect of their patients’ welfare (Gazzano *et al.* 2018). For example, cats experiencing OVH have been transported to a novel environment (possibly also via novel transport), they have undergone an operation, and they have experienced the effects of anaesthetic and also may wake up in pain in a strange and stressful place. Those veterinarians who consider this surgery routine may empathise less with these cats than with those experiencing traumatic injuries and may even underestimate the cats’ pain (Raekallio *et al.* 2003).

The interviews revealed a wide range of different perspectives among participants; for example, some expressed the view that bedding should only be used for chronically ill patients or those having undergone major surgery. Orthopaedic surgery was also mentioned as a procedure in which bedding should only be used for those patients in severe pain and not for routine surgeries such as castration and OVH. This leads us to question why cat patients undergoing major surgery are granted a ‘privilege’ which is not extended to all cat patients? The current study shows such privilege could be explained by the following factors: (i) the increased cost of major surgery is sufficient to cover the cat’s basic needs; for example, comfortable bedding; or (ii) major surgeries are relatively rare, which may lead to them being given disproportionate significance; or (iii) routine surgeries such as neutering are so commonly performed that they may lead to veterinarians experiencing detached empathy (Post *et al.* 2014). The latter may be the case, particularly where participants felt cat patients would only suffer minimal pain as a result of undergoing OVH or other routine procedures. In the field of human medicine, detached empathy is where, in the course of performing routine procedures, surgeons experience a state of detachment from the patient owing to the ‘everyday’ nature of the surgery (e.g. knee replacement) (Post *et al.* 2014). However, knee replacement, like cat neutering, is not a routine experience for the patient.

### The limitations of this study

By using a questionnaire, we were able to obtain information on the resources provided by participants to post-operative cats. However, the questionnaire was perhaps less helpful as a guide to understanding the barriers faced by participants as regards providing good cat welfare. This was because the answers provided to the questionnaire could be limited and vague. The use of a qualitative method involved conducting-semi-structured interviews enabling us to understand the social cognitive variables which might explain why post-operative cats in practices are not being provided with the basic resources they need for good welfare. However, the recruitment of participants for the semi-structured interviews displayed a substantial bias since participants were recruited by mixed invitation, either through email or Facebook messenger. This is because some participants were SSBZ’s colleagues but their participation in the present study could also be motivated by an interest in the topic or even owing to their friendship with her. However, it seems that none of these factors significantly affected their responses to the questions because this study related to conditions that all of them had experienced to some extent. The responses collected from participants were comprehensive and consistent on this topic.

In addition, the recruitment process here may include several biases. The participants had been recruited through the previous research study (Zaini *et al.* 2022) and agreed to be part of the current research study. Bias may be introduced towards individuals who either already had good attitudes towards the improvement of animal welfare in practices, or an interest in improving it (Hugonnard *et al.* 2004; Hunt *et al.* 2015; Lawson 2017). In addition, bias may have been introduced since participants were evidently those inclined to complete the questionnaire. Thus, bias from respondents and non-respondents alike potentially affected this study (Porter & Whitcomb 2005).

## Animal welfare implications and conclusion

Good knowledge is necessary but not sufficient for good cat care in veterinary practices and this impacts animal welfare. To improve cat welfare in practices, it is necessary to regard those veterinarians who have experience of managing cat care. The present study has provided insight into the barriers to undertaking good cat care management in practice and offered a number of measures that can be implemented to improve cat care management in Malaysia. Those barriers identified here include cost, skills in practice management, lack of understanding cat pain and perception of the importance of cleanliness and sterility to cat recovery. Veterinarians showed inadequate understanding regarding potential links between cats feeling stress and showing pain behaviours. Veterinarians wanted to provide a good analgesia but in order to so they need to fully recognise that stress can mask cat pain behaviours. There will continue to be significant room for improvement in cat care management until veterinarians level of understanding on the subject improves as well as their attitude towards it.

There are multiple measures which have been identified to help improve cat care management in practice and these mainly involve continuous education and an awareness of the importance of post-operative cat care among stakeholders (veterinarians, veterinary assistant/ nurse and clients) and the establishment of well-written guidelines such as SOP to follow in practice. Instead of focusing purely on education, interventions to increase post-operative cat care provision could include targeted elements that support behaviour change to overcome the barriers found here.
